# Risk assessment of avian influenza A(H5N5) virus from the first human case using the ferret model

**DOI:** 10.1128/jvi.00856-26

**Published:** 2026-08-11

**Authors:** Joanna Pulit-Penaloza, Jessica A. Belser, Nicole Brock, Troy J. Kieran, Claudia Pappas, Hui Zeng, Xiangjie Sun, Juan A. De La Cruz, Yasuko Hatta, Han Di, C. Todd Davis, Taronna R. Maines

**Affiliations:** 1 Influenza Division, NCIRD, Centers for Disease Control and Prevention 1242, Atlanta, Georgia, USA; St Jude Children's Research Hospital, Memphis, Tennessee, USA

**Keywords:** A(H5N5), influenza, ferret model, transmission, pathogenesis

## Abstract

The incursion of Eurasian-origin genotype A6 A(H5N5) virus into North America expanded the genetic diversity among North American highly pathogenic avian influenza viruses and heightened concern about zoonotic risk. Following a fatal human infection with the A(H5N5) virus A/Washington/2148/2025, viral replication was assessed in polarized human bronchial epithelial cells, and pathogenicity, transmissibility in direct contact and respiratory droplet models, and airborne virus shedding were evaluated in ferrets to inform pandemic risk assessment. A(H5N5) displayed robust replication in Calu-3 cells at 33°C and 37°C, showing kinetics and peak titers comparable to those of contemporary genotype B3.13 and D1.1 A(H5N1) viruses. In ferrets, A(H5N5) replicated efficiently in the respiratory tract, disseminated to extrapulmonary tissues, and caused fatal disease in all inoculated animals. Airborne transmission was not observed, and infrequent, low-level detection of virus in air samples paralleled that of A(H5) viruses that are not transmissible via air in ferrets. In a direct contact model, limited transmission was detected within 4 days of exposure, with evidence of lower respiratory tract replication in contact animals. These findings indicate that the A(H5N5) virus has the capacity for robust replication in an airway epithelial cell line and can cause severe systemic infection and mortality in ferrets but has not acquired adaptations for airborne spread in mammals. Collectively, these results underscore heterogeneity among clade 2.3.4.4b A(H5Nx) viruses in North America and the need for genotype-by-genotype evaluation of newly emerged viruses to understand public health risk.

**IMPORTANCE:**

The emergence of Eurasian-origin genotype A6 highly pathogenic avian influenza A(H5N5) virus in North America has increased viral diversity and raised concerns about zoonotic and pandemic risk. In this study, we evaluated the replication kinetics, pathogenesis, and transmission of A/Washington/2148/2025 A(H5N5) virus, which was isolated from the first reported human infection with this influenza virus subtype, using polarized human bronchial epithelial cells and the ferret model. The A(H5N5) virus replicated efficiently *in vitro* at temperatures representative of the upper and lower respiratory tracts and caused fatal systemic disease in inoculated ferrets. Limited transmission was observed during 4 days of direct contact. Airborne virus detection was infrequent and did not result in airborne transmission. These findings show that A(H5N5) virus can replicate robustly in mammalian cells and cause severe disease but lacks adaptations supporting efficient airborne spread, informing assessment of the pandemic risk posed by genotype A6 influenza viruses.

## INTRODUCTION

Since its initial detection in wild birds in late 2021, clade 2.3.4.4b highly pathogenic avian influenza (HPAI) A(H5) viruses have diversified extensively in North America, beginning with the introduction of the A(H5N1) A1 genotype followed by multiple reassortment events with North American avian influenza viruses that generated substantial genetic heterogeneity (1). Subsequent incursions into the Pacific and Atlantic migratory flyways included the Eurasian-origin A6 genotype of A(H5N5) viruses with a reassorted neuraminidase segment. Overall, clade 2.3.4.4b viruses have demonstrated an expanding host range, with repeated spillovers into mammalian species globally, raising concern about potential adaptation to mammalian hosts. Beginning in early 2024, A(H5N1) genotype B3.13 infections in dairy cattle were detected in multiple U.S. states, followed by multiple spillovers of the D1.1 genotype into dairy herds, further underscoring the ecological flexibility and cross-species transmission capacity of these viruses (2, 3). Notably, some A(H5N1) strains of the B3.13 genotype have shown a capacity for airborne transmission in the ferret model, a phenotype not generally observed with historical wild-type A(H5N1) viruses (4–6). In contrast, D1.1 genotype strains tested to date have not shown airborne transmission in this model, highlighting genotype-specific constraints on transmissibility (7, 8). Although available evidence indicates that most mammalian infections do not result in sustained onward transmission, continued spillovers into mammals and sporadic human infections underscore the need for continued surveillance and detailed virologic characterization to inform pandemic preparedness efforts.

In November 2025, the first confirmed human infection with avian influenza A(H5N5) was identified in the U.S., representing the first such case reported worldwide (9). The patient was an older adult with underlying health conditions who developed high fever, confusion, and worsening respiratory illness that required hospitalization and ultimately resulted in death. The person kept a mixed backyard poultry flock, and environmental samples from the property tested positive for avian influenza A(H5N5) virus. There was no evidence of person-to-person spread (10, 11). Genetic sequencing of viral RNA from the clinical specimen revealed that the virus, A/Washington/2148/2025 (WA/2148), belonged to clade 2.3.4.4b, genotype A6, with all eight gene segments of Eurasian avian origin (12).

This fatal case underscores the importance of thorough pandemic risk assessment for newly identified influenza A viruses. While genetic data provide important evidence, it does not fully define the risk to humans. A comprehensive phenotypic evaluation also requires an understanding of the capacity of the virus to replicate in human cells, cause disease, and transmit between mammals. Human respiratory tract cell cultures and the ferret model are widely used to inform preparedness efforts. A previous report of genotype A6 A(H5N5) viruses isolated from a crow and raccoon showed a lethal phenotype in ferrets and capacity for direct contact transmission between co-housed ferrets (13). Here, we assessed the replication capacity of WA/2148 A(H5N5) compared with that of other clade 2.3.4.4b viruses in polarized human bronchial epithelial cells (Calu-3). We then evaluated the pathogenicity and transmissibility of the WA/2148 virus in ferrets using both respiratory droplet exposure and direct contact models. We also collected aerosol samples from inoculated ferrets to better characterize the potential for airborne transmission in mammals.

## RESULTS

### Replication of the A(H5N5) virus in human bronchial epithelial cells

**Fig 1:**
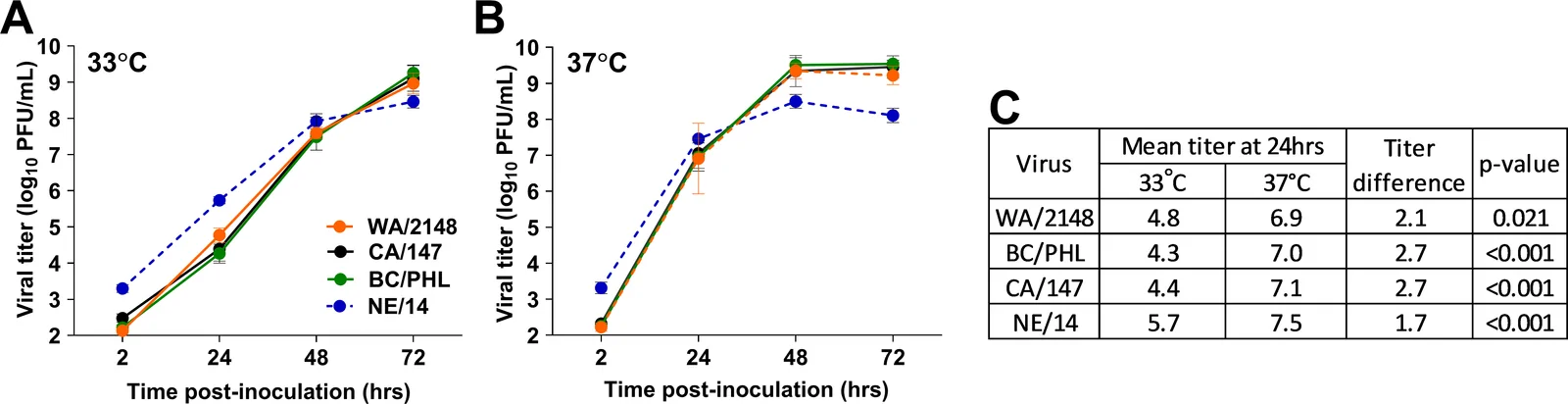
Replication kinetics of A(H5N5) virus in a polarized human airway epithelial cell line. Air–liquid interface-cultured Calu-3 cells grown on transwell inserts were inoculated apically in triplicate with 0.01 MOI of A/Washington/2148/2025 A(H5N5) (WA/2148), A/California/147/2024 A(H5N1) (CA/147), A/British Columbia/PHL-2032/2024 A(H5N1) (BC/PHL), and a human seasonal strain A/Nebraska/14/2019 A(H1N1)pdm09 (NE/14). The cells were incubated at (**A**) 33°C or (**B**) 37°C, and culture supernatants were collected at 2, 24, 48, and 72 h post-inoculation for viral titer determination by standard plaque assay. Titers are expressed as log_10_ PFU/mL ± SD. The limit of detection is 10 PFU. (**C**) Table showing mean titers (log_10_ PFU/mL), the difference between mean titers (log_10_ titer at 37°C minus log_10_ titer at 33°C), and *P* values calculated using a two-tailed equal-variance *t*-test at 24 h post-infection under the 33°C and 37°C temperature conditions.

To evaluate the ability of A(H5N5) virus to replicate productively in cells derived from the human respiratory tract relative to other A(H5) strains, Calu-3 cells cultured at the air–liquid interface were infected with WA/2148 A(H5N5), two contemporary clade 2.3.4.4b A(H5N1) viruses, A/California/147/2024 A(H5N1) (CA/147, B3.13 genotype) and A/British Columbia/PHL-2032/2024 (BC/PHL, D1.1 genotype), or a human seasonal A(H1N1)pdm09 strain, A/Nebraska/14/2019 (NE/14), at a multiplicity of infection (MOI) of 0.01. Cells were incubated at 33°C or 37°C to model temperatures of the upper and lower human respiratory tracts, respectively. All A(H5) viruses reached peak mean titers of ≥9 log_10_ PFU/mL irrespective of culture temperature. In contrast, the human seasonal virus did not exceed mean peak titers of 8.5 log_10_ PFU/mL at both temperatures (Fig. 1A and B). Area under the curve (AUC) was calculated for each time course, and the triplicate values were compared among groups using a Kruskal–Wallis test, followed by Dunn’s multiple-comparison post hoc test. At both 33°C and 37°C, no significant pairwise differences were observed between any of the tested viruses. However, replication of all tested viruses was more robust at 37°C than at 33°C, as evidenced by significantly higher titers observed at 37°C (Fig. 1C). A(H5) viruses exhibited a greater fold difference in titers between the two temperatures compared to the human seasonal virus, NE/14, consistent with previous studies showing temperature sensitivity in other A(H5) subtype viruses (14, 15). Overall, these findings indicated that A(H5N5) replicated in human epithelial cells as efficiently as other A(H5) viruses tested at these physiologically relevant temperatures.

### Pathogenesis of the A(H5N5) virus in the ferret model

**Fig 2:**
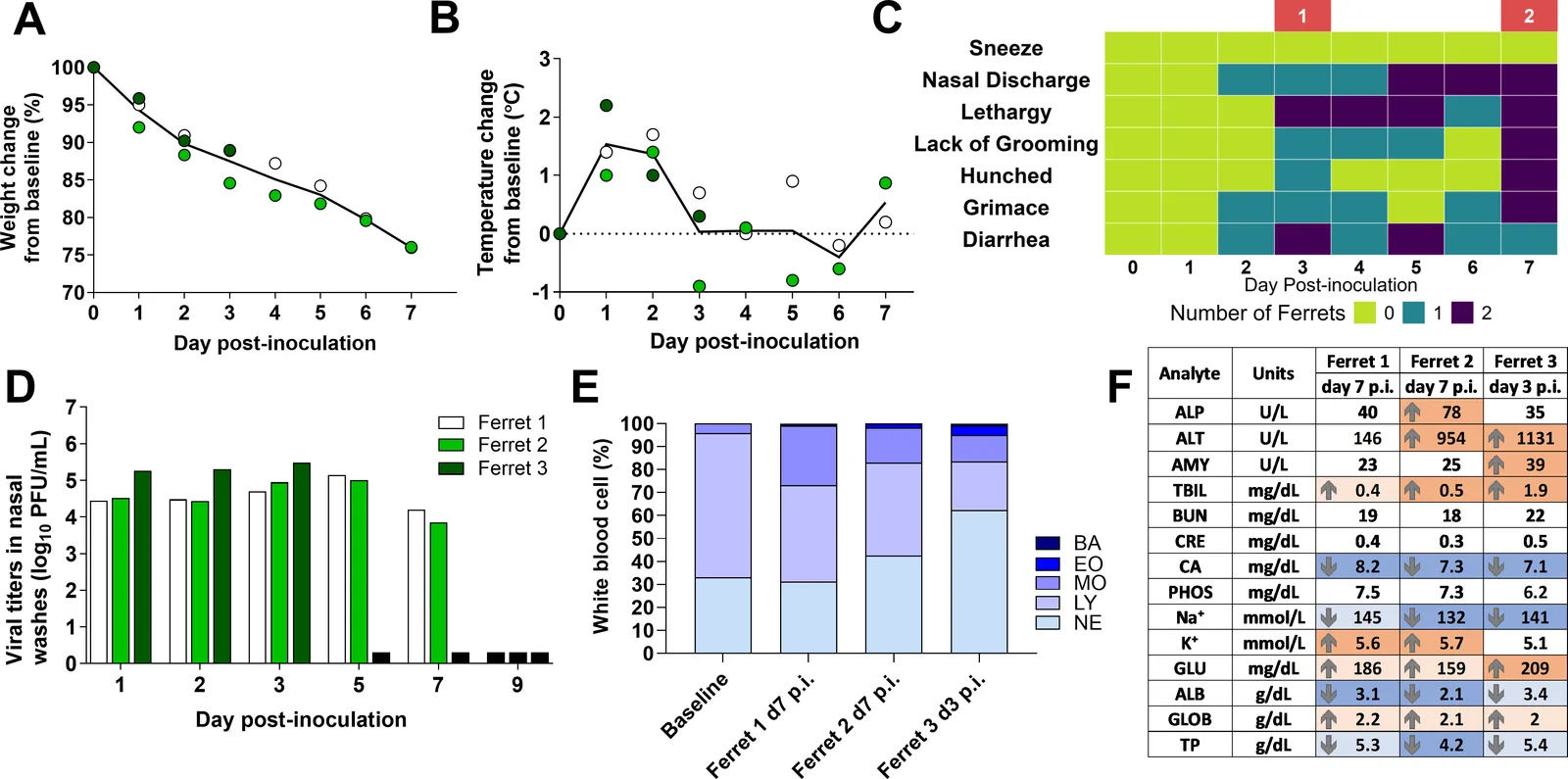
Pathogenesis of A(H5N5) virus in ferrets. Ferrets were intranasally inoculated with 6 log_10_ PFU of A/Washington/2148/2025 A(H5N5) virus (*n* = 3; ferret 1, white; ferret 2, light green; ferret 3, dark green; donors in the respiratory droplet transmission experiment). The animals were monitored for (**A**) weight loss and (**B**) temperature changes. (**C**) Clinical signs of infection were recorded on each day, and the color of each square corresponds to the number of ferrets displaying the clinical sign: lime green = 0 ferrets, teal = 1 ferret, and purple = 2 ferrets (number of ferrets euthanized on the corresponding day is shown in the red box). (**D**) Virus levels in nasal washes expressed as log_10_ PFU/mL (limit of detection 10 PFU, samples not collected following euthanasia are represented by black bars). (**E**) Whole blood collected on the day of euthanasia was used to evaluate the percentages of leukocytes (neutrophils [NE], lymphocytes [LY], monocytes [MO], eosinophils [EO], and basophils [BA]). (**F**) Serum isolated on the day of euthanasia was used to evaluate blood chemistry parameters (alkaline phosphatase [ALP], alanine aminotransferase [ALT], amylase [AMY], total bilirubin [TBIL], blood urea nitrogen [BUN], creatinine [CRE], calcium [CA], phosphorus [PHOS], sodium [Na^+^], potassium [K^+^], glucose [GLU], albumin [ALB], globulin [GLOB], and total protein [TP]). Baselines for whole blood (*n* = 18) and serum (*n* = 12) parameters were published previously (16) and are listed in Tables S1 and S2. Orange cells indicate values above baseline (up arrow), whereas blue cells indicate values below baseline (down arrow); darker shading denotes values outside the baseline ± 2× SD.

Three ferrets were intranasally inoculated with 6 log_10_ PFU of WA/2148 A(H5N5) virus and monitored to determine the kinetics of disease progression, and nasal washes were collected to evaluate virus replication in the respiratory tract. All inoculated ferrets exhibited rapid weight loss (mean maximum decrease of 19.7% from baseline), fever (mean maximum increase of 1.8°C), nasal discharge, diarrhea, and lethargy and met humane endpoint criteria between days 3 and 7 post-inoculation (p.i.) (Fig. 2A through C). A(H5N5) virus was detected in all nasal wash samples through day 7 p.i., reaching a mean maximum titer of 5.3 log_10_ PFU/mL between days 3 and 5 p.i. (Fig. 2D). On the day of euthanasia, blood was collected to assess viremia, blood cell counts (Fig. 2E), and serum chemistry parameters (Fig. 2F). These values were compared with previously published baseline values obtained from ferrets of the same sex and similar age (listed in Tables S1 and S2) (16). While total white blood cell levels from euthanized ferrets averaged 4.8 × 10⁹ cells/L, which was on the lower end of the baseline mean of 7.0 × 10⁹ cells/L (range, 3.7–10.4 × 10⁹ cells/L), lymphocyte counts were decreased, averaging 1.7 × 10⁹ cells/L, compared with a baseline mean of 4.4 × 10⁹ cells/L (range, 2.1–6.9 × 10⁹ cells/L), indicating lymphopenia. Monocyte levels were increased, averaging 0.9 × 10⁹ cells/L, compared with a baseline mean of 0.3 × 10⁹ cells/L (range, 0.1–0.8 × 10⁹ cells/L) (Table S1). While these data support that leukopenia was not pronounced, fluctuations in differential leukocyte proportions were nonetheless observed. An increase in the proportions of neutrophils, along with a decrease in lymphocytes, was most pronounced in the animals euthanized on day 3 p.i. compared with those euthanized on day 7 p.i. Absolute neutrophil counts remained within the baseline range, indicating that the observed increase reflects relative neutrophilia driven by lymphopenia rather than an absolute increase in neutrophils. Monocyte proportions were increased in all animals, particularly in those euthanized on day 7 p.i. (Fig. 2E). Markers associated with hepatic function were altered relative to baseline values (Fig. 2F). Alanine aminotransferase (ALT) levels were elevated above the reference interval in two ferrets; alkaline phosphatase (ALP) was elevated in one animal; and total bilirubin (TBIL) concentrations were elevated in all ferrets, suggesting liver-associated biochemical alterations. Changes were also observed in pancreatic tissue-associated enzymes, with amylase (AMY) elevated in one of the animals. Several electrolyte and mineral parameters were outside the reference interval. Potassium (K^+^) concentrations were elevated in two ferrets, while calcium (Ca) and sodium (Na^+^) levels were decreased relative to baseline values in all ferrets. Glucose (GLU) concentrations were elevated in all animals, indicating systemic metabolic alterations. Finally, serum protein parameters showed alterations in circulating protein fractions with total protein (TP) and albumin (ALB) levels below the reference interval, whereas globulin (GLOB) concentrations were elevated in all animals.

**Fig 3:**
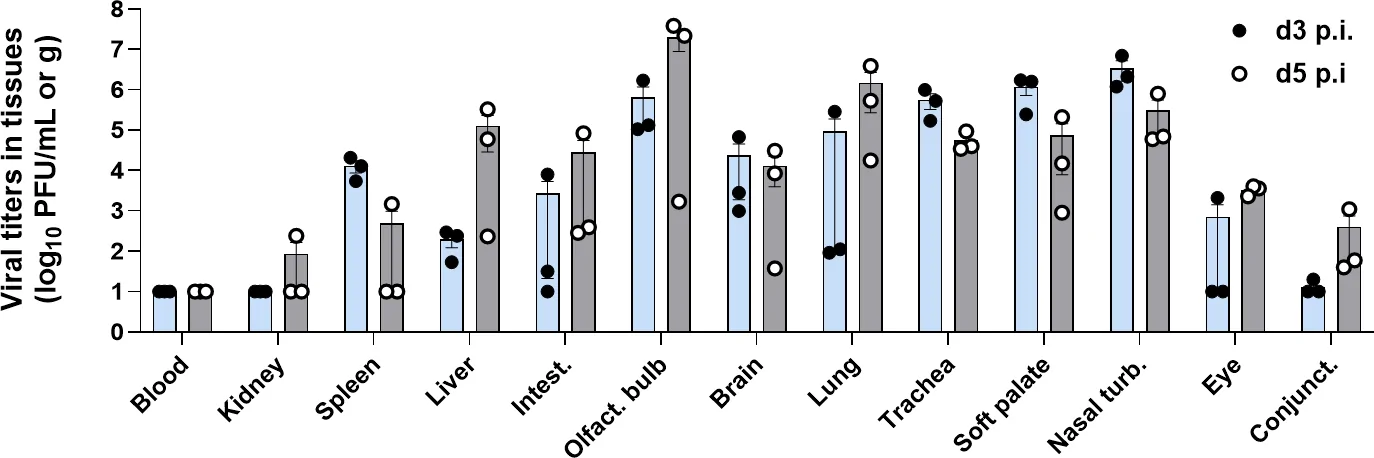
Systemic spread of A(H5N5) virus in ferrets. Ferrets (*n* = 6) were inoculated with 6 log_10_ PFU of A/Washington/2148/2025 A(H5N5) virus. Three inoculated ferrets were euthanized on day 3 post-inoculation (blue bars and black circles), and three inoculated ferrets (donors in the direct contact experiment) were euthanized on day 5 post-inoculation (grey bars and white circles) for determination of virus distribution in tissues. Mean viral titers are reported as log_10_ PFU/mL (nasal wash, blood, nasal turbinates [nasal turb.], soft palate, eye, and conjunctiva [conjunct.]) or log_10_ PFU/g (kidney, spleen, liver, intestine, olfactory bulb [olfact. bulb], brain, lung, and trachea) of tissue. The limit of detection is 10 PFU/mL or g, and negative samples were plotted at 1 log_10_ PFU/mL or g. Error bars represent the standard error of the mean. No significant differences between days 3 and 5 p.i. were observed by two-way ANOVA with Tukey’s post hoc test.

Systemic virus distribution in tissues was assessed on days 3 and 5 p.i. in six ferrets. Virus was detected throughout the respiratory tract. Systemic viral presence was evidenced by detection in numerous tissues, including the spleen, liver, intestine, olfactory bulb, brain, and ocular tissues; however, virus was not detected in the blood (Fig. 3) or rectal swabs (RSs) (data not shown) from any ferret. No significant differences in titers were found between days 3 and 5 p.i. in any of the tissues analyzed, indicating robust replication by day 3 without a significant increase in viral titers by day 5 p.i. Serum parameters in ferrets euthanized on day 5 p.i. showed patterns similar to those observed in ferrets euthanized after reaching humane endpoint criteria on days 3 and 7 p.i. (Table S2), indicative of systemic metabolic and biochemical alterations following infection.

Next-generation sequencing analysis of nasal washes and tissues collected from inoculated ferrets identified HA variants with R223S/C substitutions (227 position in H3 numbering) in many nasal wash samples and a few nasal turbinate, trachea, soft palate, and brain samples. The 223S variant was detected at a frequency greater than 50%, while the 223C variant was detected at lower frequencies (<16%), except for one brain sample, which showed 98.6% R223C substitution. These variants were not present in the virus stock inoculum. An M2 D85N variant was also observed, although the significance of this substitution is unknown. Aside from these two enriched variants, no other markers were consistently enriched across samples, and no known mammalian adaptation markers emerged in ferrets overall. In our study, the stock of the WA/2148 (egg passage 2) virus used as inoculum contained fixed PB2-627K, and this marker was maintained in the sequenced ferret samples.

### Transmission of A(H5N5) virus in ferrets

To assess whether the WA/2148 A(H5N5) virus could transmit via the airborne route, the same three ferrets monitored to determine the kinetics of disease progression (shown in Fig. 2) were used as donors in the transmission experiment. In the respiratory droplet transmission model, the inoculated ferrets were housed in cages adjacent to naive ferrets, preventing direct contact while allowing air exchange between cages. Nasal wash and rectal swab samples were collected from contact ferrets every other day until day 11 post-contact (p.c.), and no infectious virus was detected in any sample (limit of detection was 10 PFU/mL or g, data not shown). The contact ferrets did not exhibit any clinical signs of infection and did not seroconvert to the A(H5N5) virus, as evidenced by the absence of detectable antibodies in sera collected on day 21 p.c. Overall, the A(H5N5) virus did not have the capacity for airborne transmission between any of the ferret pairs.

**Fig 4:**
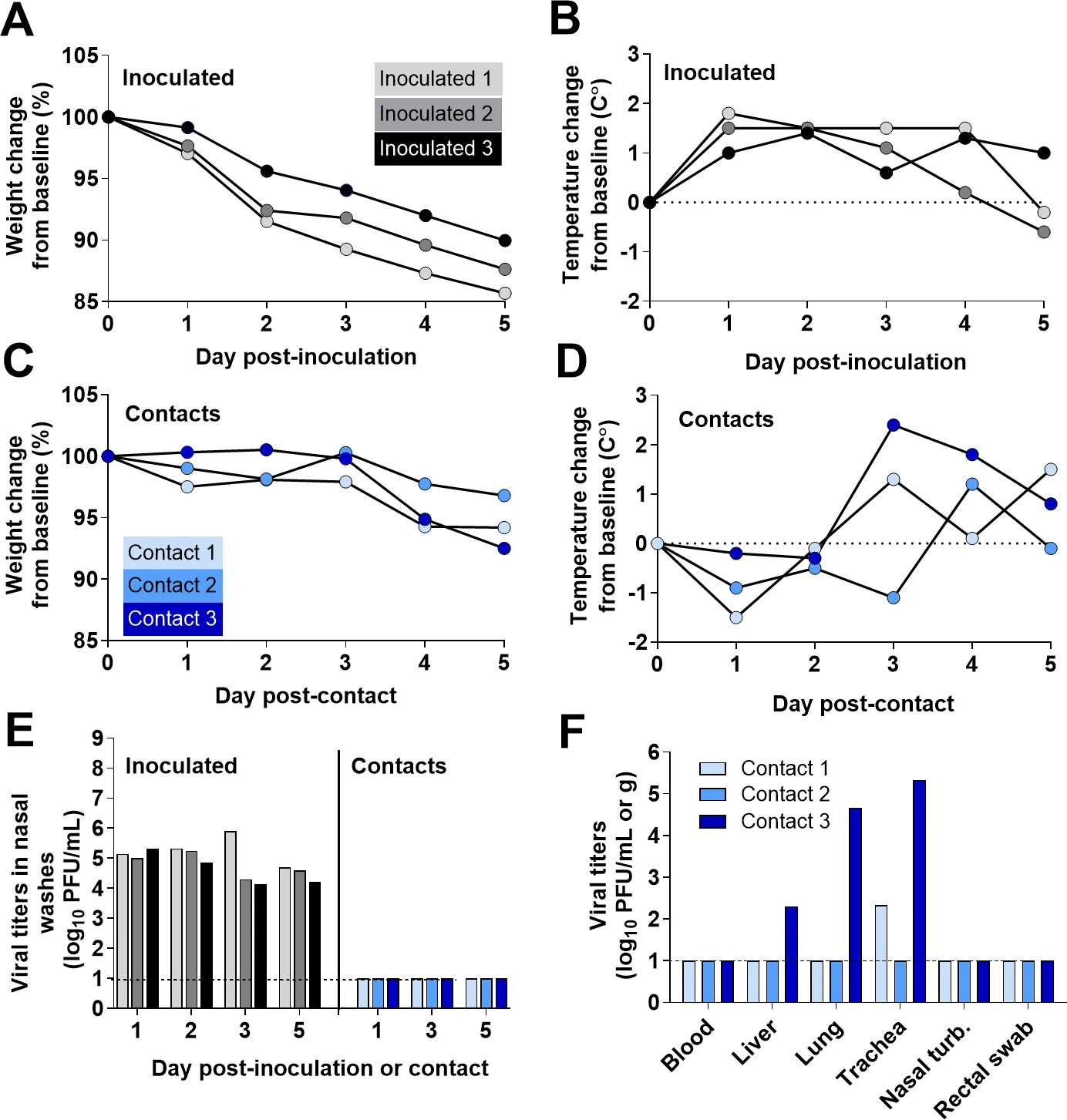
Transmission of A(H5N5) virus in ferrets in a direct contact transmission model. Transmission between three ferret pairs was assessed over a 4-day contact period, during which ferrets inoculated with 6 log_10_ PFU of A/Washington/2148/2025 A(H5N5) (gray shading) were co-housed with naive ferrets (blue shading). (**A and C**) Weight loss, (**B and D**) temperature changes, (**E**) virus levels in nasal washes, and (**F**) virus levels in tissues and rectal swabs collected from contact animals on day 5 post-contact. Viral titers are reported as log_10_ PFU/mL (nasal wash, blood, nasal turbinates [nasal turb.], and rectal swab) or log_10_ PFU/g (liver, lung, and trachea) of tissue. The limit of detection is 10 PFU/mL or g., and negative samples were plotted at 1 log_10_ PFU/mL or g.

To further examine the capacity of the A(H5N5) virus for mammalian transmission, a more permissive transmission model was used that allowed opportunity for direct, indirect, and airborne transmissions to occur. Three inoculated ferrets were co-housed with three naive ferrets at a 1:1 ratio, with contact established 24 h p.i. and maintained for 4 days, at which point the inoculated ferrets were euthanized on day 5 p.i. for determination of systemic viral spread in tissues (shown in Fig. 3). Contact animals were also euthanized on day 5 p.c. to similarly assess transmission and virus spread in tissues following close-contact exposure to A(H5N5) virus-infected ferrets. Inoculated animals exhibited comparable trends of weight loss and fever as donor ferrets in the pathogenesis assessment above, with one of three ferrets reaching humane endpoints by day 5 p.i. (Fig. 4A and B). Contact ferrets 1 and 3 displayed lethargy, fever, and weight loss of >5% of the original body weight by day 5 p.c. (Fig. 4C and D). While virus was detected in nasal washes from the inoculated ferrets at a mean maximum of 5.6 log_10_ PFU/mL between days 1 and 3 p.i., infectious virus was not recovered from any of the nasal washes collected from the contact animals (Fig. 4E). Contact ferret 1 had low levels of virus (2.3 log_10_ PFU/g) detected in the trachea, while contact ferret 3 had virus in the lung (4.4 log_10_ PFU/g), trachea (5.0 log_10_ PFU/g), and liver (2.3 log_10_ PFU/g) tissues (Fig. 4F). Overall, the data indicated that A(H5N5) has the capacity for transmission between co-housed ferrets in a close-contact model, although transmission was not robust, as only two of three contacts had detectable virus following the 4-day exposure period.

### Airborne A(H5N5) virus shedding in ferrets

**Fig 5:**
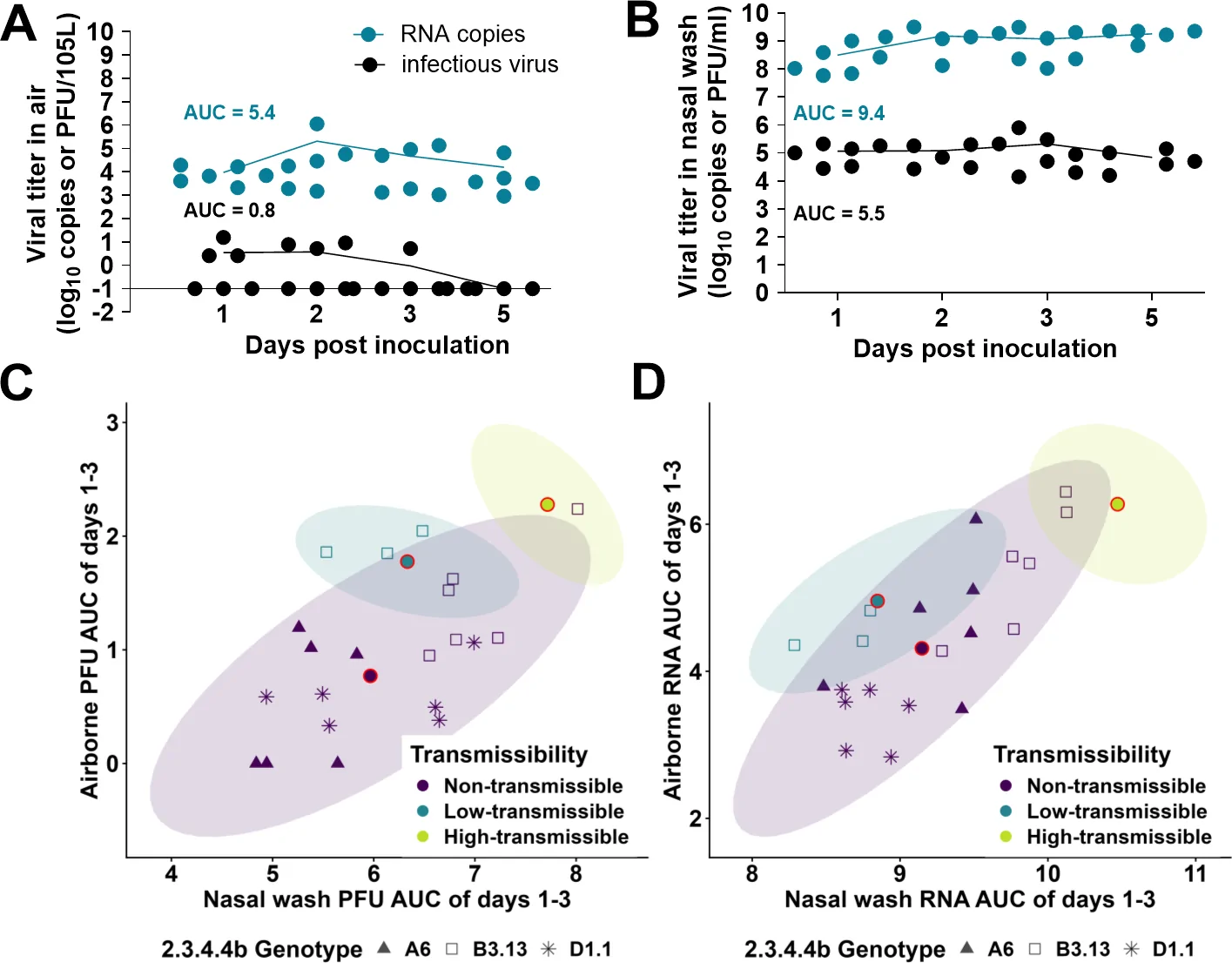
Assessment of airborne A(H5N5) virus using BC251 sampler. Air samples were collected for 30 min from ferrets inoculated with A/Washington/2148/2025 A(H5N5) (*n* = 6) using BC251 samplers. Virus titers in (**A**) air samples and (**B**) nasal washes are presented as log_10_ PFU or RNA copies per 105 L of air or mL of nasal wash. The area under the titer-versus-time curve (AUC) was calculated over days 1–3 post-inoculation and is shown as log_10_ AUC. The AUC values for infectious virus (**C**) and viral RNA copy titers (**D**) from nasal wash and air samples collected on days 1–3 post-inoculation with A/Washington/2148/2025 A(H5N5) (triangle) were contextualized with previously evaluated genotype B3.13 (square) and D.1.1 (star) clade 2.3.4.4b A(H5N1) viruses assessed using the same protocol (7). Shaded regions indicate 95% confidence intervals for previously published data for nine influenza viruses with different airborne transmissibility profiles in the ferret model: highly transmissible viruses (83%–100% transmission frequency), low-transmissible viruses (33%–50% transmission frequency), and non-transmissible (0% transmission frequency). Each symbol represents a single ferret. Red-outlined circles indicate the mean AUC for each transmissibility group. Data shown in panels C and D were previously published and are reproduced here for comparison with the newly generated A/Washington/2148/2025 A(H5N5) data (7).

Previously, we and others showed that transmissibility in the ferret model is linked to the magnitude of virus shed in nasal washes and the surrounding air (7, 17, 18). To determine whether airborne infectious virus was expelled into the environment by inoculated animals, we collected 30-min air samples from six inoculated ferrets on days 1, 2, 3, and 5 p.i. using BC251 samplers (inoculated ferrets shown in Fig. 2 and 4). Low levels of WA/2148 A(H5N5) infectious virus were sporadically detected in the air samples collected from ferrets at early time points p.i. Infectious virus and viral RNA copies were detected at the highest mean titers on day 2 p.i. at 0.6 log_10_ PFU/105 L air and 5.3 log_10_ RNA copies/105 L air, respectively (Fig. 5A). Nasal wash samples showed peak titers of 5.3 log_10_ PFU/mL on day 3 p.i. and 9.3 log_10_ RNA copies/mL on day 5 p.i. (Fig. 5B). AUC (log_10_) values of A(H5N5) infectious virus between days 1 and 3 p.i. were 0.8 for air and 5.5 for nasal wash, similar to those previously reported for A(H5) subtype viruses that do not transmit via air in the ferret model (Fig. 5C and D), and substantially lower than AUC values for A(H1N1)pdm09 and A(H9N2) viruses, which displayed highly efficient transmission via the air (7). These data further support our finding that the WA/2148 virus does not have the capacity to transmit between ferrets via the airborne route.

## DISCUSSION

The rapid expansion of clade 2.3.4.4b A(H5N1) influenza viruses into novel mammalian hosts has necessitated comprehensive risk assessments of representative viral isolates from different genotypes associated with human infection and detections in mammalian hosts (19, 20). Collectively, these studies have revealed that clade 2.3.4.4b A(H5N1) viruses can replicate efficiently in a diversity of mammalian epithelial cell types derived from the human respiratory tract (21–23), cause severe and fatal disease in animal models, and have varied capacity for airborne transmission to susceptible contacts (4–6, 18, 24). The detection of A(H5N5) virus associated with fatal human disease in 2025 underscores the confounding unpredictability of A(H5) viruses, and their continued threat to human health.

In this study, the A(H5N5) WA/2148 virus replicated efficiently in a polarized human airway epithelial Calu-3 model and exhibited *in vitro* growth characteristics similar to other recently described clade 2.3.4.4b viruses, even at the lower temperatures of the human upper airways (25). Assessment of replication kinetics using the immortalized Calu-3 cell line provides valuable context for ferret risk assessment studies. However, future studies using primary human airway epithelial cells cultured at the air–liquid interface or human airway organoids are warranted to enable a more physiologically relevant comparison of WA/2148 replication kinetics with those of human seasonal influenza viruses in the human respiratory tract. To complement these preliminary *in vitro* findings, we evaluated WA/2148 in the ferret model to further evaluate its ability to replicate in the mammalian respiratory tract as well as its pathogenicity and transmission potential *in vivo*. In ferrets, the virus also showed several features consistent with those reported for many clade 2.3.4.4b HPAI viruses, including recent North American A(H5N5) isolates (13). Severe disease with lethargy, nasal discharge, and diarrhea leading to death was observed in inoculated ferrets. WA/2148 replicated efficiently in the respiratory tract and, by day 3 p.i., had spread to extrapulmonary sites. By day 5 p.i., the virus was detected in all tissues collected, including ocular tissues, despite being undetectable in blood samples. The bloodwork findings were consistent with systemic illness at the time of euthanasia. An altered immune profile was evident, with low lymphocyte counts and relatively higher neutrophil and monocyte proportions, indicating an active inflammatory response, as previously reported in influenza-infected ferrets (26). While mild influenza A virus infection in ferrets may be associated with transient lymphopenia during the acute phase of infection, sustained and pronounced lymphopenia is typically associated with severe and fatal infection. The serum chemistry results indicated abnormalities consistent with liver dysfunction, signs of broad metabolic disturbance, and electrolyte imbalance. Lower albumin and total protein, together with higher globulin, indicated an active immune response. These findings are in agreement with prior studies of A(H5N1) virus infection in ferrets that showed elevated serum chemistry of liver enzymes, glucose, and other analytes in animals reaching humane endpoints (27–29). While lymphohematopoietic parameters are only infrequently quantified during standard risk assessments in ferrets, they provide meaningful clinical information to understand the scope of possible systemic abnormalities post-infection and areas of commonality across genetically diverse viruses associated with similar severe disease outcomes.

The transmission phenotype of WA/2148 was more restricted compared to recent A(H5N1) strains shown to have the capacity for airborne spread between ferrets and was more representative of the majority of historical and contemporary A(H5) viruses that are non-transmissible via the air in the ferret model (30, 31). The lack of airborne transmission was also supported by the low frequency and titers of virus detected in air samples collected from inoculated ferrets. Previous studies have linked ferret transmissibility to the magnitude of virus shed in nasal washes and into the surrounding air (7, 17, 18). In those studies, transmissible viruses were generally shed into the air more frequently and at higher levels than non-transmissible viruses, and nasal wash titers also tended to be higher, though exceptions were noted. Here, we calculated the AUC for the A(H5N5) virus and compared it with previous data on virus shedding in the air measured in ferrets over the first 3 days after inoculation with nine viruses spanning multiple subtypes, differing host origins, and transmissibility profiles (7). WA/2148 virus showed AUC values similar to those reported for A(H5) viruses not capable of airborne transmission in the ferret model, further supporting the absence of airborne transmission in our study.

Since A(H5N5) WA/2148 virus was unable to transmit via the air, we evaluated transmission in a less stringent model by co-housing the inoculated ferrets with naive ferrets to test transmission in the presence of direct contact. Transmission models utilizing shortened or intermittent exposures can more closely emulate human exposure conditions (32, 33). In this study, a modified transmission model was used where direct contact was limited to 4 days. Within this time frame, virus was recovered at necropsy from the tissues of two ferrets that showed clinical signs of illness, despite the absence of detectable virus shedding in nasal washes. These data indicated that the virus can be transmitted between ferrets in close contact, and the virus preferentially replicated in the lower respiratory tract. Similar observations were made with closely related clade A(H5N5) viruses isolated from a crow and a raccoon in North America. Although the virus was not recovered from nasal washes of co-housed contacts, severe disease necessitating euthanasia was observed (13). Lack of detectable virus in nasal washes despite evidence of productive infection has also been reported for other A(H5) viruses (7, 34). Overall, the limited transmission of WA/2148 virus in the modified direct contact transmission setting is in agreement with transmission patterns observed with historical and contemporary A(H5) subtype viruses in ferrets (24, 30, 31, 35–37), though it is possible that increased frequency of transmission via this mode may have been detected if donor ferrets had remained in constant exposure to contact animals until all donor ferrets met humane endpoints.

Mutational frequencies observed in the ferret model may provide insights into the transmission potential of zoonotic viruses in humans (38). Previous analyses of samples collected from ferrets inoculated with clade 2.3.4.4b A(H5N1) viruses revealed no major genetic changes at known mammalian adaptation marker locations, suggesting a lack of strong selective pressure against these viruses (5–7, 24). In this study, HA 223S/223C variants were detected across multiple ferret samples, whereas these variants were not detected in the inoculum or the original isolate (11). As HA residue 223 lies adjacent to the receptor binding site, enrichment of 223S/C variants may indicate functional selection *in vivo*. However, R223S and R223C are not established markers of mammalian adaptation, in contrast to S223N, which has been shown to increase binding to α2,6-linked receptors (39–41). Therefore, the relevance of the HA R223S/C variants identified in ferret samples remains unclear. Analysis of all available genotype A6 HA sequences in GISAID showed that every consensus sequence encoded 223R, indicating that these substitutions are not commonly observed in circulating viruses. While the original human clinical specimen contained a mixed 627E/K population, with 627E predominating (11), the stock of the WA/2148 virus used as inoculum in our study (egg passage 2) contained fixed PB2-627K. This substitution has been associated with enhanced replication in the mammalian respiratory tract and increased transmissibility in ferrets (41). The genotype A6 viruses A/Raccoon/PEI/FAV-0193-1/2023 and A/American_Crow/PEI/FAV-0035-6/2023, which were previously studied in ferrets, did not possess this mutation. However, this marker has been detected in other North American and Eurasian A(H5N5) viruses sequenced from both mammals and birds (13). Analysis of available GISAID clade 2.3.4.4b A6 genotype viruses from the Americas and Eurasia indicates that approximately 34% have PB2 627K (data not shown). Of these, 87% of the viruses were sequenced from birds, indicating that this change likely does not reflect continual emergence of this mammalian adaptive marker as a result of mammalian infections but rather is a persistent marker consistently found in many wild bird hosts (42). Emergence of additional known mammalian adaptation markers in ferrets was not observed in this study.

Overall, the WA/2148 A(H5N5) virus showed the capacity to cause severe, systemic mammalian infection and robust replication in polarized human airway epithelial cells, characteristics similar to those of other contemporary A(H5Nx) viruses. However, the virus lacked clear adaptation for efficient airborne spread, indicating limited adaptation to a mammalian host. Our findings underscore substantial heterogeneity in pathogenesis and transmission among clade 2.3.4.4b A(H5Nx) viruses, supporting continued genotype-by-genotype evaluations.

## MATERIALS AND METHODS

### Biosafety

All experiments involving HPAI viruses were conducted under enhanced biosafety level 3 enhanced containment, following safety protocols established by the U.S. Department of Agriculture and the Select Agent Program, in accordance with Biosafety in Microbiological and Biomedical Laboratories guidelines (43).

### Viruses

Virus stocks of A/British Columbia/PHL-2032/2024 A(H5N1) (BC/PHL) and A/Nebraska/14/2019 A(H1N1)pdm09 (NE/14) were propagated in MDCK cells at 37°C for 48 h. WA/2148 and CA/147 were propagated in the allantoic cavities of 10-day-old embryonated chicken eggs incubated at 37°C for 24–26 h. Allantoic fluid from multiple eggs was pooled; supernatants were clarified by centrifugation, aliquoted, and stored at –80°C (44). Viral titers were determined by standard plaque assay, and full-genome sequencing was performed to confirm subtype identity and exclude contamination with other influenza strains.

### Replication in Calu-3 cells

Calu-3 cells (ATCC) were seeded on 12-well plate semipermeable membrane inserts (0.4 µm pore size, Corning) as previously described (45) until reaching a transepithelial resistance of >1,000 Ω^2^, then the apical media was removed, and cells were differentiated at the air–liquid interface for 2 weeks. Cells were infected at an MOI of 0.01 for 1 h at 37°C or 33°C, with cells returned to respective temperature-specific incubators following removal of inoculum and washing of the apical surface. At each indicated time point, 200 µL of serum-free media was added to the apical surface for 15 min prior to collection and storage at −80°C. Samples were titered in MDCK cells for determination of infectious titer by standard plaque assay.

### Ferrets

Healthy, castrated, male Fitch ferrets (*n* = 12; age, 8–9 months old; Triple F Farms, Sayre, PA) were used for this study. All ferrets were seronegative for circulating influenza A(H1N1) and A(H3N2) and B viruses, as confirmed by hemagglutination inhibition assay (44). Ferrets were housed inside Duo-Flo Bioclean mobile units (Lab Products Inc., Seaford, DE, USA; 150–180 air changes/h) during experiments. Ferrets were anesthetized before transponder placement, virus inoculation, blood collection, nasal and rectal sample collection, and euthanasia. Anesthesia was administered intramuscularly using a combination of 10–25 mg/kg ketamine (100 mg/mL ketamine HCl) and 2 mg/kg xylazine (100 mg/mL xylazine HCl). Nine naive ferrets were intranasally inoculated with 6 log_10_ PFU of virus in 1 mL of phosphate-buffered saline (PBS), split between both nares. Three ferrets were euthanized on day 3 p.i. for determination of virus distribution in tissues. Three ferrets were used as donors in the direct contact transmission experiment until day 5 p.i. and then euthanized for determination of virus distribution in tissues. Three ferrets were used as donors in the respiratory droplet transmission experiment and were monitored to determine the kinetics of disease progression and survival. Lethargy was scored as previously described to calculate a relative inactivity index (8): 0, alert and playful; 1, alert and playful only when stimulated; 2, alert and not playful when stimulated; and 3, neither alert nor playful when stimulated. Animals exhibiting persistent severe illness (e.g., persistent lethargy, diarrhea, labored breathing, and ≥25% weight loss) were sedated, then humanely euthanized via cardiac administration of 1.0 mL/kg, 390 mg pentobarbital sodium, and 50 mg phenytoin sodium per 1 mL. Prior to euthanasia, 1 mL of blood was collected via venipuncture of the cranial vena cava. Blood (250 µL) was immediately transferred to a 1.5 mL K_2_EDTA microtainer tube (BD, Franklin Lakes, NJ, USA) and gently inverted, while 750 µL was transferred to a Vacutainer Rapid Serum Tube (BD). All blood and serum samples were processed and analyzed within 1 h of collection. Nasal washes were performed by flushing the nares with 1.0 mL of sterile PBS–BSA solution (0.1% BSA, 100 U/mL penicillin, 100 U/mL streptomycin, and 50 µg/mL gentamycin). RSs were obtained using a sterile cotton-tipped applicator, moistened with sterile PBS–BSA solution (0.1% BSA, 200 U/mL penicillin, 200 U/mL streptomycin, and 100 µg/mL gentamycin), inserted approximately 1–2 inches into the rectum, and then placed into a tube containing 1 mL of sterile PBS–BSA solution. The samples were stored at –80°C until titration using a standard plaque assay.

### Hematological and serological assays

Complete blood cells were quantified from blood collected in a 1.5 mL K_2_EDTA microtainer tube using a Hemavet HV950FS instrument per the manufacturer’s instructions (Drew Scientific, Inc., Oxford, CT). The hematology parameters included white blood cell count (10^9^ cells/L), neutrophils (10^9^ cells/L, %), lymphocytes (10^9^ cells/L, %), monocytes (10^9^ cells/L, %), eosinophils (10^9^ cells/L, %), basophils (10^9^ cells/L, %), red blood cell count (10^12^ cells/L), hemoglobin (g/dL), hematocrit (%), mean corpuscular volume (fL), mean corpuscular hemoglobin (pg), mean corpuscular hemoglobin concentration (g/dL), red cell distribution width (%), platelet count (10^9^ cells/mL), and mean platelet volume (fL). Serum was analyzed using a VetScan VS2 Chemistry Analyzer (Zoetis, Parsippany, NJ, USA) and Comprehensive Diagnostic Profile rotors (Abaxis, Union City, CA, USA), per the manufacturer’s instructions. The clinical chemistry parameters included albumin (g/dL), alkaline phosphatase (U/L), alanine aminotransferase (U/L), amylase (U/L), total bilirubin (mg/dL), blood urea nitrogen (mg/dL), calcium (mg/dL), and phosphorus (mg/dL), creatinine (mg/dL), glucose (mg/dL), sodium (mmol/L), potassium (mmol/L), total protein (g/dL), and globulin (g/dL). Baseline levels are shown in Tables S1 and S2.

### Aerosol collections

Awake, inoculated ferrets (*n* = 6) were individually placed in sanitized, ventilated plastic transport containers (23.9 L, solid-walled with a perforated lid) as previously described (7). Air was sampled for 30 min using the BC251 (National Institute for Occupational Safety and Health) sampler, which collected air at 3.5 L/min (105 L of air in 30 min) and separated aerosols into >4 µm, 1–4 µm, and <1 µm fractions (46). Samplers and containers were disinfected with 70% ethanol, rinsed with water, and air-dried after use. Most of the viral RNA was detected in the >4 µm size fraction, in agreement with previous findings (17); therefore, total combined titers are reported. Each sample (140 µL) was inactivated in AVL buffer (QIAGEN, Hilden, Germany) and stored at −80°C for RNA quantification. The remaining volume was kept on ice and used for virus quantification by plaque assay in MDCK cells within 2 h of sampling. Viral RNA was extracted from the samples using the QIAamp Viral RNA Mini Kit (QIAGEN), with 140 µL of sample used for each extraction. We conducted real-time RT-PCR in duplicate using the SuperScript III Platinum One-Step qRT-PCR System (Invitrogen, Waltham, MA, USA), employing a primer and probe set specific to the influenza A virus M1 gene (17). A standard curve created with samples of known M gene copy numbers was used to extrapolate the RNA copy numbers for the influenza virus M.

### Next-generation sequencing

Viral RNA was reverse transcribed and whole-genome amplified using universal influenza primers (47) with Superscript III One-Step RT-PCR Platinum Taq High Fidelity Kit (Invitrogen) in a 12.5 µL reaction following the manufacturer’s protocol. The PCR product was verified on a 2% agarose E-Gel with SYBR Safe (Invitrogen) and purified with Sera-Mag SpeedBeads (Fisher Scientific, Pittsburgh, PA, USA). Next-generation sequencing was performed on purified amplicons using the Illumina Nextera DNA Flex library preparation kit (Illumina, San Diego, CA, USA) at half-volume reaction size. Libraries were quality checked and quantified on a TapeStation 4150 (Agilent Technologies, Santa Clara, CA, USA) and pooled in equal molar ratio prior to sequencing on Illumina iSeq100 (Illumina) with i1 Reagent Cartridge v2 using paired-end 150 bp reads. Data were processed in Geneious Prime v2023.0.4, trimming reads with BBduk v38.84 to remove primers, adapters, and quality below Q30, and keeping reads with a minimum length of 40 bp. Trimmed reads were then mapped to the reference/inoculum genome with 50 bp N spacers between gene segments. Variants were called at 5% frequency using a minimum coverage of 100× and excluding paired reads mapped more than 30% from the expected distance of 500 bp.

### Statistical analysis and data presentation

*In vitro* results were analyzed by calculating *P* values using a two-tailed equal-variance *t*-test at 24 h post-infection under the 33°C and 37°C temperature conditions. AUC was calculated for each biological replicate for each virus using the trapezoidal rule across all measured time points. PFU per milliliter values were log_10_-transformed prior to AUC calculation. AUCs were compared among groups using a Kruskal–Wallis test separately for 33°C and 37°C, followed by Dunn’s multiple-comparison post hoc test (*P* values adjusted for multiple testing). Differences in tissue titers were evaluated using two-way ANOVA with Tukey’s post hoc test. Figures were assembled using GraphPad Prism v10.5.0. Graphs 2C, 5C, and 5D were made using R. For each virus, ferret, and sample type, the AUC was calculated for PFU and RNA across days 1–3 using the trapezoidal method on the untransformed values transformed using the DescTools v0.99.60 R package (48), and the resulting AUCs were then log_10_-transformed. To visualize the relationship between nasal wash and airborne virus levels across transmissibility categories, log_10_ AUC values were plotted against one another, with group means and 95% confidence ellipses overlaid for each category and points shaped by clade 2.3.4.4b genotype using tidyverse v2.0.0 (49), ggplot2 v4.0.3 (50), and ragg v 1.5.2 (51) R packages in R v4.5.3 (52).

## Data Availability

Next-generation sequencing data have been deposited in the NCBI Sequence Read Archive (PRJNA1473223). The WA/2148 stock sequence was uploaded to GISAID: EPI_ISL_20450317. The code used to generate figures in R and all data used for the figures has been deposited at GitHub: https://github.com/Troy-Kieran/Risk-assessment-of-avian-influenza-A-H5N5-virus-from-the-first-human-case-using-the-ferret-model/tree/main.

## SUPPLEMENTAL MATERIAL

Supplemental material jvi.00856-26-s0001.docx Tables S1 and S2.

ASM does not own the copyrights to Supplemental Material that may be linked to, or accessed through, an article. The authors have granted ASM a non-exclusive, world-wide license to publish the Supplemental Material files. Please contact the corresponding author directly for reuse.
