# Supplementary material for: Risk assessment of avian influenza A(H5N5) virus from the first human case using the ferret model
Source: J Virol. 2026 Aug 11;100(9):e00856-26. doi: 10.1128/jvi.00856-26 (PMC13595979; doi:10.1128/jvi.00856-26)
Supplement: Supplementary file 1 [file jvi.00856-26-s0001.docx]

**Supplemental material for:**

**Risk assessment of avian influenza A(H5N5) virus from the first human case using the ferret model**

Joanna Pulit-Penaloza^1*^, Jessica A. Belser^1^, Nicole Brock^1^, Troy J. Kieran^1^, Claudia Pappas^1^, Hui Zeng^1^, Xiangjie Sun^1^, Juan A. De La Cruz^1^, Yasuko Hatta^1^, Han Di^1^, C. Todd Davis^1^, Taronna R. Maines^1*^

**Affiliations**

^1^Influenza Division, NCIRD, Centers for Disease Control and Prevention, Atlanta, GA, USA

**Table 1. Summary of hematological parameters in ferrets inoculated with A(H5N5) virus.**

| **Analyte^a^** | **Units** | **Baseline^b^** | **Day of euthanasia** | | |
| --- | --- | --- | --- | --- | --- |
|  |  |  | **7** | **7** | **3** |
| WBC | 10^9^ cells/L | 7.0 (3.7-10.4) | 4.84 | 5.64 | 3.88 |
| NEU | 10^9^ cells/L | 2.3 (1.1-4.1) | 1.51 | 2.4 | 2.42 |
| LYM | 10^9^ cells/L | 4.4 (2.1-6.9) | **2.04** | 2.28 | **0.81** |
| MON | 10^9^ cells/L | 0.3 (0.1-0.8) | **1.25** | **0.85** | 0.45 |
| EOS | 10^9^ cells/L | <0.1 | 0.04 | 0.09 | 0.16 |
| BAS | 10^9^ cells/L | <0.1 | 0.1 | 0.01 | 0.03 |
| NEU | % | 33.0 (16.6-54.7) | 31.19 | 42.59 | 62.44 |
| LYM | % | 62.9 (44.5-76.1) | 42.05 | 40.46 | 21 |
| MON | % | 4.1 (0.8-8.5) | 25.8 | 15.15 | 11.61 |
| EOS | % | <0.1 | 0.73 | 1.66 | 4.2 |
| BAS | % | <0.1 | 0.22 | 0.17 | 0.74 |
| RBC | 10^12^ cells/L | 95 (7.9-10.5) | **11.75** | **11.08** | **11.14** |
| HGB | g/dL | 14.0 (11.6-16.0) | **18.4** | **18.4** | **17.9** |
| HCT | % | 44.7 (38.6-49.2) | **49.5** | 46.8 | 46.3 |
| MCV | fL | 47.1 (46.0-50.0) | **42.5** | **42.2** | **41.6** |
| MCH | pg | 14.7 (14.0-15.5) | **15.7** | **16.6** | **16.1** |
| MCHC | g/dL | 31.2 (29.0-32.9) | **37.2** | **39.3** | **38.7** |
| RDW | % | 18.56 (17.4-19.3) | **17.3** | **17.3** | **16.8** |
| PLT | 10^9^ cells/L | 389.6 (43.9-573) | 244 | 300 | 244 |
| MPV | fL | 9.0 (7.5-14.2) | 10.7 | 10 | 11.2 |

Ferrets were intranasally inoculated with 6 log10 PFU of A/Washington/2148/2025 A(H5N5) virus and were euthanized on day 3 or 7 post inoculation (n=3).

^a^ Whole blood collected on the day of euthanasia was evaluated; White Blood Cell (WBC), Neutrophils (NEU), Lymphocytes (LYM), Monocytes (MON), Eosinophils (EOS), Basophils (BAS), Red Blood Cells (RBC), Hemoglobin (HGB), Hematocrit (HCT), Mean Corpuscular Volume (MCV), Mean Corpuscular Hemoglobin (MCH), Mean Corpuscular Hemoglobin Concentration (MCHC), Red Cell Distribution Width (RDW), Platelet Count (PLT), and Mean Platelet Volume (MPV).

^b^ Baseline range represents the mean, with the lower (2.5%) and upper (97.5%) limits of the reference interval (RI) shown in parentheses. Baselines for each parameter were published previously (n=18) (1).

Boldface denotes values that are outside baseline RI.

**Table 2. Summary of serum analytes in ferrets inoculated with A(H5N5) virus.**

| **Analyte^a^** | **Units** | **Baseline^b^** | **STDEV** | **2xSTDEV** | **Ferret1** | **Ferret2** | **Ferret3** |
| --- | --- | --- | --- | --- | --- | --- | --- |
| ALP | U/L | 32.3(13.2-42.8) | 10.79 | 21.5845 | 29 | **46** | 24 |
| ALT | U/L | 171.7(72.7-348.1) | 93.01 | 186.017 | **904** | **1400** | **873** |
| AMY | U/L | 19.5(10.5-26.3) | 5.36 | 10.7186 | **62** | **47** | **35** |
| TBIL | mg/dL | 0.2(0.1-0.3) | 0.07 | 0.13371 | **0.5** | **0.6** | **0.5** |
| BUN | mg/dL | 32.5(18.0-43.2) | 9.21 | 18.4194 | 25 | 41 | **13** |
| CRE | mg/dL | 0.6(0.3-0.9) | 0.24 | 0.47065 | 0.3 | 0.5 | 0.3 |
| CA | mg/dL | 9.7(9.3-10.4) | 0.33 | 0.66856 | **6.8** | **6.7** | **7.6** |
| PHOS | mg/dL | 6.9(5.8-8.3) | 0.76 | 1.51558 | 8 | **9.6** | 6.5 |
| Na⁺ | mmol/L | 151.3(147.3-155.0) | 2.63 | 5.26567 | **137** | **136** | **134** |
| K⁺ | mmol/L | 5.1(4.9-5.2) | 0.11 | 0.21909 | **6.3** | **6.4** | **5.8** |
| GLU | mg/dL | 114.4(79.8-153.7) | 26.91 | 53.8243 | **170** | **188** | **195** |
| ALB | g/dL | 4.3 (4.0-4.9) | 0.29 | 0.58672 | **2** | **1.9** | **2.2** |
| GLOB | g/dL | 1.7(1.4-2.0) | 0.201 | 0.40113 | **2.6** | 1.7 | **2.3** |
| TP | g/dL | 6.1(5.5-6.9) | 0.438 | 0.87525 | **4.5** | **3.7** | **4.5** |

Ferrets were intranasally inoculated with 6 log10 PFU of A/Washington/2148/2025 A(H5N5) virus and were euthanized on day 5 post inoculation (n=3).
^a^ Serum isolated on the day of euthanasia was used to evaluate blood chemistry parameters [Alkaline Phosphatase (ALP), Alanine Aminotransferase (ALT), Amylase (AMY), Total Bilirubin (TBIL), Blood Urea Nitrogen (BUN), Creatinine (CRE), Calcium (CA), and Phosphorus (PHOS), Sodium (Na+), Potassium (K+), Glucose (GLU), Albumin (ALB), Globulin (GLOB) and Total protein (TP)].

^b^ Baseline range represents the mean, with the lower (2.5%) and upper (97.5%) limits of the reference interval (RI) shown in the parentheses.

Baselines for each analyte were established and published previously (n=12) (1).

Boldface denotes values that are outside baseline RI.

References

1.	Velazquez EM, Basu Thakur P, Brock N, Maines TR, Belser JA. 2025. Establishment and validation of a ferret model for systemic antibiotic treatment during influenza A virus infection. Lab Anim (NY) 54:188-194.
